# Interference of functional dual-tasks on gait in untrained people with Parkinson’s disease and healthy controls: a cross-sectional study

**DOI:** 10.1186/s12891-020-03431-x

**Published:** 2020-06-22

**Authors:** Constanza San Martín Valenzuela, Lirios Dueñas Moscardó, Juan López-Pascual, Pilar Serra-Añó, José M. Tomás

**Affiliations:** 1 Unit of Personal Autonomy, Dependency and Mental Disorder Assessment, Faculty of Medicine, Blasco Ibáñez, 15, 46010 Valencia, Spain; 2 Department of Physiotherapy, Faculty of Physiotherapy, University of Valencia, Gascó Oliag Street, 5, 46010 Valencia, Spain; 3 UBIC Reseach Group, Department of Physiotherapy, Faculty of Physiotherapy, University of Valencia, Gascó Oliag Street, 5, 46010 Valencia, Spain; 4 Centro Investigación Biomédica en Red de Salud Mental, CIBERSAM, Av. Monforte de Lemos, 3-5, 28029 Madrid, Spain; 5 Biomechanics Institute of Valencia, Polytechnic University of Valencia, Camino de Vera, s/n, 46022 Valencia, Spain; 6 Department of Behavioral Sciences Methodology, Faculty of Psychology, University of Valencia, Blasco Ibánez Avenue, 13, 46010 Valencia, Spain

**Keywords:** Functional dual-task, Biomechanical gait analysis, Kinematics, Kinetic, Parkinson’s disease

## Abstract

**Background:**

In Parkinson’s disease (PD) population, performing secondary tasks while walking further deteriorates gait and restrict mobility in functional contexts of daily life. This study (1) analyzed the interference of functional cognitive and motor secondary task on untrained people with PD and (2) compared their walking with healthy subjects.

**Methods:**

Forty people with PD (aged 66.72 [7.5] years, Hoehn and Yahr stage I-II-III, on-medication) composed the PD group (PDG) and 43 participants (aged 66.60 [8.75] years) formed the group of healthy counterparts (HG). Gait was evaluated through spatiotemporal, kinematic and kinetic outcomes in five conditions: single task (ST) and visual, verbal, auditory and motor dual-task (DT).

**Results:**

The velocity, stride length, and braking force performance of both groups was statistically higher in the ST condition than in verbal, auditory and motor DT (*p* < .05), and inferior in double support time and midstance force (p < .05). The same pattern was observed when compared the ST and visual DT condition, where participants showed a significantly higher stride length, double support time and braking force in the ST (*p* < .05). In addition, the PDG exhibited a significant shorter double support time and midstance force, and showed a higher braking force in the visual DT than in the verbal DT (*p* < .05). Similarly, the PDG showed a wider stride in the visual DT than in the motor DT condition (p < .05). PDG participants had a significantly lower performance than the HG in all the variables analyzed except for the maximum hip extension in the stance phase (*p* > .05). *Conclusions*: In untrained participants with PD, verbal and motor secondary tasks affect gait significantly, while auditory and visual tasks interfere to a lesser extent. Untrained people with PD have a poorer gait performance than their healthy counterparts, but in different grades according to the analyzed variables.

**Trial registration:**

The data in this paper are part of a single-blind, randomized, controlled trial and correspond to the evaluations performed before a physical rehabilitation program, retrospectively registered with the number at clinicaltrial.govNCT04038866.

## Background

Parkinson’s disease (PD) is a chronic, progressive and neurodegenerative pathology, with motor and non-motor disorders [1], which affects functional mobility [2]. Still, people with PD report that gait impairments are the most disabling motor symptoms of the disease [3]. For this reason, several studies have characterized gait in people with PD, observing a deterioration of spatiotemporal variables, such as velocity, stride length, cadence and double support time [4, 5].

However, walking in everyday situations is carried out simultaneously with other activities. This is known as dual-tasking (DT) and involves the development of two tasks with different objectives at the same time [6], where attention is placed in one of the tasks or alternated between the primary task (gait) and the secondary task (i.e. cognitive or motor). In the PD population, performing secondary tasks while walking further deteriorates gait [4] and restricts walking in functional contexts of daily life.

Despite the amount of published information regarding how DT affects gait in PD, so far as we know, these studies do not include kinematic and kinetic variables [5] simultaneously to the spatiotemporals, thus preventing a comprehensive characterization of the gait pattern or comparisons with a control group. Also, the majority of studies stress the importance of the complexity of the secondary task [4] and use elaborate activities in the evaluation that allow them to easily see a change in gait such as mathematical calculations. But there is no consensus on what kind of secondary task could interfere most [7] (e.g. visual versus verbal), and daily secondary tasks have not been explored.

Gait assessment with secondary functional tasks, such as talking with another person or carrying something with the hands, would allow observing the performance of people with PD in everyday contexts and guiding physical therapy with the aim of preparing patients for these probable challenges. This is of special interest in PD because the basal ganglia are involved in the control of different aspects besides motor control [8, 9] and the onset of cognitive impairment can further impair the performance of the inevitable daily dual-tasks.

Based on the foregoing, the general objective of the present study was to analyze the degree of interference of functional cognitive and motor secondary tasks on the walking biomechanics in untrained people with Parkinson’s disease. Likewise, a secondary aim of the study was to compare a parkinsonian walking pattern with gait in healthy older people during the execution of single and dual-tasks.

## Material and methods

### Study design and participants

The study design was cross-sectional with a convenience sample (specifically, modal instance sampling) and adhered to the STROBE guidelines (http://www.strobe-statement.org/). Eighty-three participants integrated the sample, of which 40 people with Parkinson’s disease composed the PD group (PDG) and 43 participants formed the group of healthy counterparts (HG) matched by age, gender, and height. PDG participants were recruited from two centers: a Parkinson’s disease Association and the Neurology Service of a local public hospital. HG individuals were from a Municipal Senior Social Activity Center. Inclusion criteria for PDG were: diagnosis of idiopathic PD and to present Hoehn & Yahr stadium I, II or III. In addition, both groups should be able to walk by themselves, have a normal cognitive state (i.e. score > 25) according to the Minimental test adapted for PD [10], symmetry in lower limb length (< 1 cm), and at least two months of sedentary life and without having attended physiotherapy sessions Exclusion criteria for both groups were the presence of another neurological or symptomatic musculoskeletal disease (e.g. musculoskeletal pain), history of trauma or surgery on the lower limbs, balance disorders due to other diseases and uncontrolled chronic diseases (e.g. hypertension or diabetes).

### Procedures

The assessments were carried out at the Medicine Department of the University of Valencia. PDG participants were evaluated in the on-medication state [4]. The assessment session included first, a clinical interview to record the main personal data of the participants and to verify their cognitive state; second, an anthropometric evaluation to record weight, height, and length of lower limbs [11] used in the standardization of biomechanical variables; and third, biomechanical assessment of gait at a self-selected comfortable speed. All the participants walked barefoot under all conditions tested to avoid the damping provided by the different footwear of the participants and therefore, prevent a confusing variable in obtaining ground reaction forces. Five *conditions* were randomly evaluated: i. single task (ST): walking without secondary tasks with the attention focused only on walking performance, ii. visual DT: walking while checking the time on an analog clock projected at the end of the walkway, iii. Verbal DT: walking while telling the evaluator the activities they had performed the previous day in chronological order, iv. auditory DT: walking while listening and recognizing different daily noises and, v. motor DT: walking while carrying one glass in each hand and repeatedly transferring their contents from one to the other (beans). These tasks were intended to simulate the action of looking at the time, talking with another person, listening to the sounds of the environment and manipulating objects with hands while walking. All these tasks are representatives of daily life activities, which provide external validity to the study and concur with skills previously studied [4, 12]. During DT gait, participants were urged to focus attention on the secondary task through all the way walking.

### Biomechanical gait assessment

Gait assessment was carried out in a corridor 10 m long and data were registered using 3D photogrammetry with 12 smart cams (Kinescan/IBV software, Biomechanical Institute of Valencia, Valencia, Spain, version 5.3.0.1) and two force platforms (Dinascan/IBV Biomechanical Institute of Valencia, Valencia, Spain). A valid repetition is one in which the patient performs at least five complete strides along the corridor and where one complete and isolated footprint (rigth or left) from the third stride coincide with the dynamometric platform located in the center of the corridor. The previous and subsequent strides were discarded to avoid acceleration and deceleration phases, at the beginning and at the end of the gait, respectively. For each of the five evaluated walking *conditions*, 10 repetitions were performed, five with each foot, to later use the average of these. Participants were allowed to rest between repetitions, sitting on a stool for less than three minutes, when fatigue was reported.

The biomechanical model was composed of 35 landmarks located in specific anatomical points on both sides of the body: i. the spinous process of the seventh cervical vertebra, ii. the acromioclavicular joint, iii. The posterosuperior iliac spine, iv. the anterior-superior iliac spine, v. the greater trochanter of the femur, vi. the anterior vertex triangle forming the thigh segment, vi. the medial condyle of the knee, vii. The lateral condyle of the knee, viii. The posterior vertex triangle of the leg segment, ix. the medial malleolus of the ankle, x. lateral malleolus of the ankle, xi. the posterior surface of calcaneus, xii. The tuberosity of fifth metatarsal and xiii. Cuboids. For suitable biomechanical modeling, the patient’s clothing included shorts, sleeveless shirt and slip-on paddings (with the top of the foot uncovered). Before recording gait, participants were allowed to walk in the corridor (ST condition) to familiarize themselves with the test.

### Outcomes

For calculation of biomechanical variables based on the data exported from the photogrammetry system and the dynamometric platform, the software was programmed with Matlab (MathWorks, MA, USA, version R2016b). The average of these dependent variables was calculated from the 10 repetitions for each condition: i. Velocity: distance travelled by the body per unit of time (m/s), ii. Stride length: distance measured between two consecutive heel strikes of the same foot (i.e. two steps) (m), iii. Cadence: number of steps taken in a minute (steps/min), iv. Double support time: the sum of the amount of time in which there is double-limb support in a gait cycle (%), v. Ankle range: range of motion (ROM) that corresponds to the sum of the maximum angle of plantar flexion and maximum angle of dorsiflexion of the foot (°), vi. Hip flexion: maximum flexion angle reached by the hip joint during the swing phase of the gait cycle (°), vii. Hip extension: maximum extension angle reached by the hip joint during the stance phase of the gait cycle (°), viii. Weight-acceptance force: first peak of the vertical component curve of reaction forces corresponding to the maximal weight-acceptance (N), ix. Midstance force: minimum value between the two force peaks of the vertical component curve of reaction forces corresponding to the midstance of the gait cycle (N), x. Braking force: first negative peak of anteroposterior component curve of reaction force that corresponds to the braking (N).

In addition, to inform about the degree of interference of dual tasks during gait, the Dual-task cost was calculated as the percentage measure of performance decline observed under DT *conditions* (Eq. 1) [4, 13]. Negative values mean a decrease in the value measured during the dual-task compared with the single-task condition. Likewise, the same equation was used to describe the performance of PDG with respect to the HG participants, which was defined as the Performance of parkinsonian gait (PPgait). When the PPgait percentage (Eq. 2) was negative, it meant that the PDG had registered lower values in the variable analyzed than the HG, while positive PPgait indicated that the PDG has recorded higher values than the HG. For the calculation of both equations, the mean values obtained in each outcome and group were used.

$$ DT\  cost\ \left(\%\right)=\frac{\left( ST\  score- DT\  score\right)}{ST\  score}\times 100 $$

Equation 1: Dual-task cost. Interference rate of dual tasks during gait.

$$ PPgait\left(\%\right)=\frac{\left( EPG\  score- HG\  score\right)}{EPG\  score}\times 100 $$

Equation 2: Performance of parkinsonian gait. Impairment of the group with Parkinson’s disease compared to the healthy group.

### Data analyses

Statistical analyses were performed using IBM SPSS v.24 (SPSS Inc., Chicago, IL, USA). Standard statistical methods were used to obtain the mean and standard deviation (SD). Also, as descriptive results, the confidence interval, PPgait percentage, and DT cost were calculated. A two-factor mixed Multivariate analysis of variance was conducted to analyze the effect of within-subject factor *conditions* with five categories (ST, visual DT, verbal DT, auditory DT, and motor DT) and between-subject factor *group* with two categories (i.e. PDG and HG) on the dependent biomechanics variables. Some of these variables were standardized according to anthropometric data, concretely, ‘stride length’, which was standardized by the lower limb length, and ground reaction force variables were standardized according to the weight of the participants.

When significant factor effects were found, the Bonferroni correction, provided by the statistics package, was used for pairwise comparisons. Differences were declared statistically significant if *p* < 0.05 and the 95% confidence interval (CI) of the pairwise mean differences was reported.

To check for differences between the demographic outcomes between groups, multivariate analysis with one-way between-subject factor *group* was conducted. Further, to demonstrate differences in gender between groups, a chi-square test was used.

## Results

### Participants

Eighty-three participants completed this study. Their characteristics are shown in Table 1. No significant differences were observed between groups in age, weight, height, body mass index (BMI), leg length or gender (*p* > .05). Throughout the evaluation, the PD participants did not present disorientation in any of the dual-task cognitive conditions or apparent loss of memory during the verbal dual-task condition.

**Table 1:** Clinical and demographical variables of participants

	PDG		HG		Between-groups
	Range	Mean (SD)	Range	Mean (SD)	CI (95%)
Age (years)	44–79	66.72 (7.50)	43–83	66.60 (8.75)	−2.98 to 4.69
Weight (kg)	43–99	70.45 (12.13)	50–103	68.43 (12.15)	−6.24 to 4.97
Height (m)	1.44–1.76	1.61 (.07)	1.42–1.82	1.58 (.08)	−.07 to .00
BMI	16.37–39.57	26.51 (4.63)	21.93–38.77	27.22 (4.00)	−1.28 to 2.69
Leg length (m)	78–97	85.84 (4.52)	73–99	85.34 (4.99)	−3.31 to 1.20
Evolution (years)	1–23	5.78 (4.67)	NP	NP	
	**Frequency**	**(%)**	**Frequency**	**(%)**	***p*-value**
Hoehn & Yahr scale	I:4	10	NA	NA	–
	II:9	22.5	NA	NA	–
	III:27	67.5	NA	NA	–
Gender	Male: 17	42.5	Male: 15	34.88	.47
	Female: 23	57.5	Female: 28	65.12	

Main demographic data for Parkinson’s disease group (PDG) and healthy control (HG). Range, mean, standard deviation (SD) and frequency are shown. NA: not applicable. BMI: body mass index. Differences between groups from multivariate analysis with one-way between-subject factor *group* are reported with 95% CI and the difference between Gender from chi-square test are indicated by the *p* value (statistically significant differences *p* < .05). * Indicates statistically significant effects.

The interaction effect between *condition* and *group* factor was statistically significant (*p* < .05) on double support time, ankle range and midstance force outcomes (Table 2). This indicates that the gait patterns of both groups varied in a different way across the conditions evaluated. For the rest of the outcomes analyzed, the factors had an isolated effect (p < .05), indicating that the patterns of velocity, stride length, cadence, hip flexion, weight-acceptance and breaking forces were similar for both groups through the conditions evaluated (Table 2). On the maximum hip extension variable, no significant effect was observed (*p* > .05).

**Table 2:** Main factors effects and their interactions on outcomes measures

Outcomes	Condition	Group	Condition * Group
Velocity	*F*_(2.26; 183.66)_ = 15.98; *p* = .00; ƞ^2^_p_ = .17 ^*^	*F*_(1; 81)_ = 50.88; *p* = .00; ƞ^2^_p_ = .39 ^*^	*F*_(2.26; 183.66)_ = .33; *p* = .85; ƞ^2^_*p*_ = .04
Stride length	*F*_(2.70; 218.93)_ = 18.46; *p* = .00; ƞ^2^_p_ = .19 ^*^	*F*_(1; 81)_ = 28.07; *p* = .00; ƞ^2^_p_ = .26 ^*^	*F*_(2.70; 218.93)_ = 1.40; *p* = .24; ƞ^2^_p_ = .02
Cadence	*F*_(2.71; 219.95)_ = 4.50; *p* = .00; ƞ^2^_p_ = .05 ^*^	*F*_(1; 81)_ = 7.79; *p* = .00; ƞ^2^_p_ = .10 ^*^	*F*_(2.71; 219.95)_ = .64; *p* = .57; ƞ^2^_p_ = .00
Double support time	*F*_(3.66; 296.66)_ = 24.81; *p* = .00; ƞ^2^_p_ = .23 ^*^	*F*_(1; 81)_ = 42.35; *p* = .00; ƞ^2^_p_ = .34 ^*^	*F*_(3.66; 296.66)_ = 2.58; *p* = .04; ƞ^2^_p_ = .03 ^*^
Ankle range	*F*_(3.47; 281.12)_ = 5.54; *p* = .00; ƞ^2^_p_ = .06 ^*^	*F*_(1; 81)_ = 12.24; *p* = .00; ƞ^2^_p_ = .13 ^*^	*F*_(3.47; 281.12)_ = 3.15; *p* = .01; ƞ^2^_*p*_ = .04 ^*^
Hip extension	*F*_(2.73; 221.63)_ = 2.27; *p* = .08; ƞ^2^_p_ = .03	*F*_(1; 81)_ = .32; *p* = .56; ƞ^2^_*p*_ = .00	*F*_(2.73; 221.63)_ = 1.05; *p* = .36; ƞ^2^_p_ = .01
Hip flexion	*F*_(3.50; 283.80)_ = 8.57; *p* = .00; ƞ^2^_p_ = .10 ^*^	*F*_(1; 81)_ = 6.89; *p* = .01; ƞ^2^_p_ = .10 ^*^	*F*_(3.50; 283.80)_ = 1.67; *p* = .16; ƞ^2^_p_ = .02
Weight-acceptance force	*F*_(3.25; 263.55)_ = 7.04; *p* = .00; ƞ^2^_p_ = .10 ^*^	*F*_(1; 81)_ = 10.87; *p* = .00; ƞ^2^_p_ = .12 ^*^	*F*_(3.25; 263.55)_ = 2.23; *p* = .08; ƞ^2^_p_ = .03
Midstance force	*F*_(3.58; 290.46)_ = 26.69; *p* = .00; ƞ^2^_p_ = .25 ^*^	*F*_(1; 81)_ = 24.96; *p* = .00; ƞ^2^_p_ = .24 ^*^	*F*_(3.58; 290.46)_ = 2.54; *p* = .04; ƞ^2^_p_ = .03 ^*^
Braking force	*F*_(2.98; 241.49)_ = 17.38; *p* = .00; ƞ^2^_p_ = .18 ^*^	*F*_(1; 81)_ = 28.34; *p* = .00; ƞ^2^_p_ = .26 ^*^	*F*_(2.98; 241.49)_ = 2.31; *p* = .07; ƞ^2^_p_ = .03

For each effect, the value of the F ratio (*F*), the number of degrees of freedom of factor and error, the *p*-value and the eta square are shown. * Indicates statistically significant effects (*p* < .05).

### Dual-task interference during gait

**Fig. 1:**
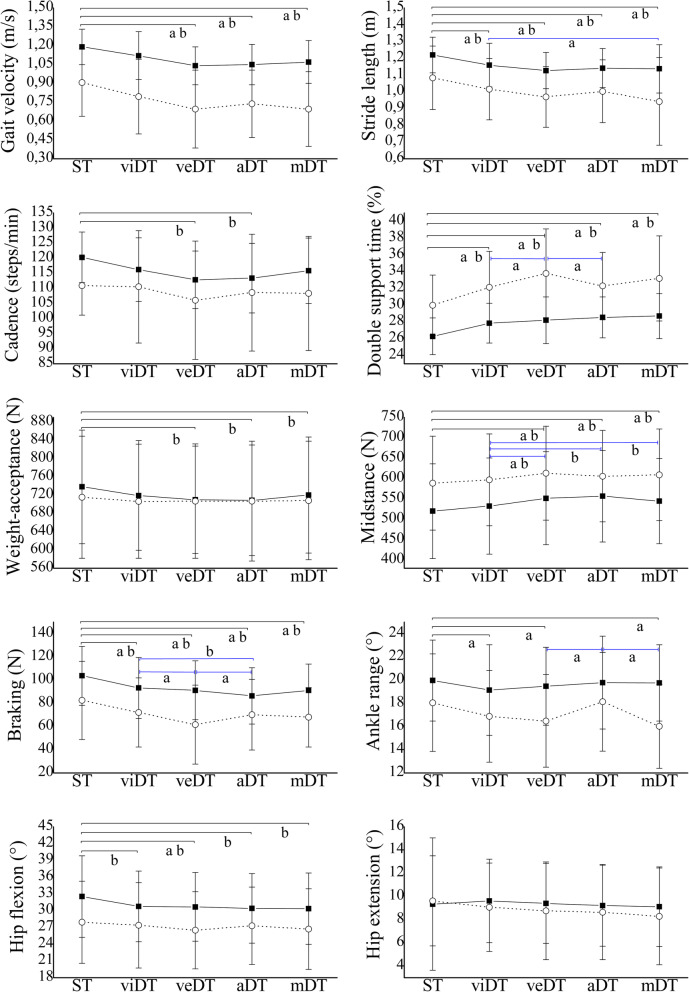
**Gait performance of both groups in all conditions of study.** Mean and standard deviation represented with the vertical lines for the Parkinson’s disease group (PDG, dashed line) and the healthy group (HG, continuous line) for the Single-task (ST), visual (viDT), verbal (veDT), auditory (aDT) and motor (mDT) dual-task conditions. The upper horizontal black lines represent the statistical differences between single-task and dual-task conditions, while the differences between dual conditions are represented in blue. The letters below each horizontal line indicate statistically significant differences of the Parkinson’s disease group (a) and statistically significant differences of the healthy group (b)

Post-hoc analyses showed that the performance of both groups for the ST condition was higher than visual DT (p < .05) only in stride length, double support time and braking force (Fig. 1), which represented a DT cost for PDG of 6.19, − 7.16% and 12.59%, respectively (Table 3). In addition, the same behavior with respect to these two conditions was observed in PDG participants, who had a significant decrease in range of ankle motion during the visual DT (DT cost: 6.48%), while HG participants showed a deterioration in hip flexion outcome (*p* < .05). In velocity, cadence and both vertical forces measured, the performance of participants was similar in both, visual DT and ST condition (*p* > .05).

**Table 3:** Dual-task cost

Outcomes	Condition to compare	PDG		HG	
		DT cost	95% CI	DT cost	95% CI
Velocity	Visual	−7.49	−.04 to .19	− 6.09	−.04 to .18
	Verbal	−14.97	.07 to .22*	−12.70	.08 to .22*
	Auditory	−12.20	.06 to .17*	−12.19	.09 to .20*
	Motor	−15.09	.08 to .20*	−10.34	.06 to .18*
Stride length	Visual	−6.19	.01 to .14*	−4.95	.01 to .13*
	Verbal	−10.48	.07 to .18*	−7.61	.05 to .15*
	Auditory	−7.40	.03 to .15*	−6.39	.03 to .14*
	Motor	−13.0	.08 to .25*	−6.74	.01 to .18*
Cadence	Visual	−.32	−5.12 to 5.82	−3.37	−1.22 to 9.33
	Verbal	−4.33	−1.12 to 10.73	−6.19	1.73 to 13.16*
	Auditory	−2.07	−4.06 to 8.65	−5.72	.75 to 13.01*
	Motor	−2.28	−3.04 to 8.10	−3.68	−.94 to 9.80
Double support time	Visual	7.16	−3.39 to −.89*	5.96	−2.77 to −.36*
	Verbal	12.63	−5.25 to −2.32*	7.30	−3.33 to −.50*
	Auditory	7.70	−3.28 to −1.32*	8.56	−3.19 to − 1.30*
	Motor	10.66	−4.57 to −1.81*	9.19	−3.74 to −1.08*
Ankle range	Visual	−6.48	.00 to 2.33*	−4.16	−.29 to 1.95
	Verbal	−8.68	.51 to 2.61*	−2.47	−.51 to 1.50
	Auditory	.45	−1.66 to 1.50	−.93	−1.34 to 1.71
	Motor	−11.24	.59 to 3.46*	−.99	−1.18 to 1.57
Hip extension	Visual	−5.70	−2.07 to .98	3.01	−1.19 to 1.75
	Verbal	−8.87	−2.35 to .64	.61	−1.39 to 1.50
	Auditory	−10.01	−2.40 to .47	−1.47	− 1.52 to 1.25
	Motor	−13.84	−2.97 to .30	− 2.37	− 1.80 to 1.36
Hip flexion	Visual	−1.94	−1.13 to 2.22	−5.44	.15 to 3.39*
	Verbal	−5.15	.17 to 2.69*	−5.66	.62 to 3.05*
	Auditory	−2.23	−.78 to 2.03	−6.51	.75 to 3.47*
	Motor	−4.37	−.03 to 2.47	− 6.71	.97 to 3.39*
Weight-acceptance force	Visual	−1.24	−.19 to .39	−2.55	−.00 to .56
	Verbal	−1.20	−.11 to .35	−3.80	.19 to .65*
	Auditory	−1.18	−.13 to .38	− 3.95	.19 to .69*
	Motor	−.99	−.17 to .39	−2.42	.01 to .56*
Midstance force	Visual	1.37	−.34 to .09	2.42	−.40 to .02
	Verbal	4.16	−.58 to −.11*	6.09	−.70 to −.24*
	Auditory	2.91	−.49 to −.01*	7.02	−.78 to −.31*
	Motor	3.47	−.54 to −.05*	4.75	−.63 to −.16*
Braking force	Visual	−12.59	−.24 to −.04*	− 10.03	−.25 to −.05*
	Verbal	−25.30	−.42 to −.17*	− 11.96	−.30 to −.06*
	Auditory	− 14.82	−.29 to −.05*	− 16.67	−.37 to −.14*
	Motor	− 17.59	−.39 to −.04*	− 11.97	−.36 to −.02*

Differences between single-task and dual-tasks conditions for each group from post-hoc analysis are shown with de 95% confidence interval (95% CI). * Indicates significant statistical differences between single-task and dual-task condition (p < .05). The dual-task cost was calculated with eq. 1. Negative percentages mean a decrease in the value measured during the dual-task compared with the single-task condition

Regarding the verbal, auditory and motor dual-tasks, a significant worsening of gait velocity, stride length, double support time, midstance and braking forces were observed in both groups (*p* < .05), shown in Fig. 1 and Table 3. In fact, the highest DT cost % of both, PDG and HG, were observed in these outcomes, mainly for verbal and motor tasks (Table 3), reaching in the PDG participants a verbal DT cost of 25% during the performance of the braking force. Besides these variables, in the weight-acceptance force and cadence, we observed a deterioration in the dual conditions mentioned only for HG (p < .05), whereas the PDG presented a deterioration of the ankle range under these same dual-tasks. With respect to maximum hip flexion, although in the HG a decrease of the angle was observed in all dual conditions (*p* < .05), in the PDG only this decrease was observed during the verbal DT condition (p < .05).

In addition, significant differences between dual conditions were observed, mainly between the visual DT and another dual-task. Specifically, PDG performance was significantly better during the visual DT condition than the motor DT on stride length (mean difference: .08; 95% CI = .00 to .17) and the verbal DT condition on double support time (mean difference: 1.64; 95% CI = − 3.06 to −.21), midstance (mean difference: 2.27; 95% CI = −.41 to −.03) and braking forces (mean difference: .15; 95% CI **=** −.28 to −.02). Also, we observed that the performance of the PDG during verbal DT was significantly worse than the auditory DT condition (*p* < .05) in double support time (mean difference: 1.47; 95% CI **=** .30 to 2.65), ankle range (mean difference: 1.64; 95% CI **=** − 3.16 to −.13) and braking force (mean difference: .12; 95% CI **=** .01 to .22) outcomes. In a different way, HG showed differences between dual conditions chiefly in the midstance force, where they had worse performance with verbal (mean difference: .28; 95% CI **=** −.46 to −.10), auditory (mean difference: .36; 95% CI **=** −.53 to −.18) and motor (mean difference: .20; 95% CI **=** −.40 to −.01) tasks compared to the visual task.

### Differences between groups for each condition

The differences between post-hoc analysis pairs of measures are shown in Table 4. The PDG participants had a significantly worse performance than HG in all conditions on gait velocity, stride length, double support time, ankle range, hip flexion and midstance, weight-acceptance and braking forces (p < .05). Conversely, a similar hip extension gait pattern was observed for all conditions evaluated and, besides, on cadence and ankle range during auditory DT condition (*p* > .05).

**Table 4:** Gait performance differences between groups

Outcomes	Condition	PPgait%	95% CI
Velocity	Single	−22.03	.14 to .29*
	Visual	−23.88	.13 to .30*
	Verbal	− 25.29	.13 to .29*
	Auditory	−22.04	.11 to .27*
	Motor	−28.86	.16 to .32*
Stride length	Single	−12.60	.1 to .25*
	Visual	−14.09	.1 to .26*
	Verbal	−16.21	.12 to .27*
	Auditory	−13.82	.1 to .25*
	Motor	−20.78	.14 to .35*
Cadence	Single	−8.48	5.44 to 13.36*
	Visual	−5.16	.85 to 12.25*
	Verbal	−6.37	.1 to 13.24*
	Auditory	−4.43	−2.1 to 11.72
	Motor	− 6.92	.83 to 14.18*
Double support time	Single	12.38	−5.0 to −2.42*
	Visual	13.37	−5.8 to −3.76*
	Verbal	16.53	−7.41 to −3.75*
	Auditory	11.68	−5.21 to −2.33*
	Motor	13.55	−6.26 to −2.73*
Ankle range	Single	−10.62	.23 to 3.60*
	Visual	−13.36	.54 to 4.00*
	Verbal	−18.13	1.4 to 4.60*
	Auditory	−9.10	−.15 to 3.45
	Motor	−23.38	2.24 to 5.25*
Hip extension	Single	2.84	−1.88 to 2.42
	Visual	−6.13	−2.18 to 1.10
	Verbal	−7.27	−2.32 to 1.05
	Auditory	−6.39	−2.21 to 1.11
	Motor	−10.09	−2.5 to .82
Hip flexion	Single	−16.49	1.4 to 7.80*
	Visual	−12.33	.32 to 6.43*
	Verbal	−15.86	1.35 to 7.05*
	Auditory	−11.39	.24 to 6.00*
	Motor	−13.63	.67 to 6.61*
Weight-acceptance force	Single	−3.17	.29 to 1.06*
	Visual	−1.80	.11 to .89*
	Verbal	−0.46	.10 to .65*
	Auditory	−0.28	.05 to .66*
	Motor	−1.68	.28 to .77*
Midstance force	Single	11.66	−1.09 to −.46*
	Visual	10.75	−1.03 to −.39*
	Verbal	10.03	−.92 to −.39*
	Auditory	8.13	−.75 to −.21*
	Motor	10.57	−.96 to −.40*
Braking force	Single	−25.66	−.53 to −.18*
	Visual	−29.35	−.51 to −.17*
	Verbal	−48.10	−.64 to −.29*
	Auditory	−22.93	−.43 to −.12*
	Motor	−34.23	−.50 to −.26*

Differences between groups for each condition evaluated from post-hoc analysis are shown with the 95% confidence interval (95% CI). * Indicates significant statistical differences between groups (p < .05). PPgait: Performance of parkinsonian gait (%), negative values mean Parkinson’s disease group had registered lower values in the variable analyzed than the healthy group

These differences between PDG and HG are reflected in the PPgait%, whose value changes according to the analyzed variables. PDG participants walk slower and perform a lower braking force, by more than 20% in relation to the performance of HG participants. This deficit drops between 15 and 10% approximately in the stride length, double support time, ankle range and midstance force outcomes.

## Discussion

This study analyzed how functional dual tasks affect parkinsonian gait and compared walking performance of people with Parkinson’s disease with healthy older adults under single and dual-task. To our knowledge, this is the first study that uses functional dual-task in parkinsonian gait assessment and analyzes kinematic and kinetic parameters, in addition to spatiotemporal variables, which are the most used in previous studies [4, 14].

Regarding dual-task interference, the gait of participants with PD and healthy controls significantly deteriorated when performing a dual-task but, this impairment depended on the type of secondary task and outcomes analyzed. First of all, we observed that the visual secondary task showed the least interference on gait in both groups, since this was the only condition in which all study participants showed a similar gait pattern to ST condition, specifically in gait velocity, cadence and both vertical forces. Indeed, both groups showed a better walking with a visual task than with other secondary tasks, although the differences between dual tasks were more evident in the PDG. To explain why visual tasks may interfere less during gait compared to other secondary tasks is complex due to sight is a common resource for performing an appropriate walking pattern [15], consequently, a vision interruption should be negative on the gait of participants with PD. It is possible that visual tasks are more common in daily life during gait performance and therefore, the participants were more accustomed to this type of dual-task. On the other hand, the type of visual task used in this study (to observe different times of an analog clock projected on the wall at the end of the walkway), allowed subjects to have a sufficiently wide field of view. The visual tasks that are close to the face or approach the visual objects to the subject (such as looking at the mobile or the time on a wristwatch) reduce the visual field, which would deteriorate the balance. Conversely, to observe forward objects, allows to get information about the environment in which we walk, that is what occurred in our visual DT. Supporting this theory, it was observed in previous studies that having a reduced visual field in people with glaucoma worsens the balance [16], as well as in people with PD when they face a dynamic visual field [17].

By contrast, the secondary task that most interfered with gait was not the same for both groups. For HG, the interference caused by verbal, auditory and motor secondary tasks was similar between the measured outcomes, that is, no significant differences were observed between the dual conditions beyond the differences observed with visual tasks and the percentages of DT cost are similar between them.

Nevertheless, participants with PD showed the most interference on gait with the verbal and motor secondary tasks, specifically in the outcomes where the interaction of the factors analyzed in this study was statistically significant (double support time, ankle range and midstance force), besides of braking force. That is to say, the PD participants achieved a better gait with a visual or auditory task than with verbal or motor secondary tasks.

The verbal tasks could be more complex to perform than other secondary tasks due to the subcomponents involved: lexicon, message planning, message extension and oral motor movement. From there, the motor part of speech produces the largest interference in the context of the dual-task paradigm [18], so it could be using resources from other cortical areas that are hyper-activated in parkinsonian gait, as a compensation for the lack basal ganglia movement regulation [19] such as the frontal lobe [20]. Likewise, basal ganglia have direct control over the motor function [8]; accordingly, two concurrent motor tasks could be competing for the same neural resources [4], which would explain the high interference of the motor task with the arms. Future studies should differentiate the benefits of gait rehabilitation with high-interference and low-interference secondary task. Moreover, the progression of gait exercises that starts with secondary visual tasks that allow having a wide visual field should be considered.

Concerning the second objective of this study, the results showed that the performance of PDG is significantly lower than the performance by HG, in all the outcomes except for hip extension during the stance phase of the gait cycle. However, the lower performance of the PDG was not always the same for all the variables, which was evidenced by the PPgait percentage. While in velocity and braking force the PPgait percentage was greater than 20%, in the stride length, double support time, ankle range, hip flexion and midstance force outcomes, the PPgait percentage varied between approximately 10 and 15%. On the other hand, a performance below or around 5% was observed in cadence and weight-acceptance force.

In general, it seems interesting to note that PPgait is especially high during the motor condition when observing most of the variables analyzed, reaching a 34% deficit in the braking force while developing the motor DT. This could indicate that there are aspects of Parkinsonian gait that are more affected than others and that the context of the gait could influence it as well. An example of this is that people with PD have difficulty to take large steps while the ability to control cadence is maintained or deteriorates more progressively [21, 22].

Further, the results of the kinematic variables are in consonance with these results. We have observed that participants with PD only differed from control participants in the ankle range and hip flexion, achieving a similar maximum hip extension. Other authors have previously observed a decreased plantar flexion in the first stages of PD (Hoehn & Yahr II) [5], while in more advanced stages (Hoehn & Yahr II.V and III), alterations of ankle dorsiflexion would further reduce ankle ROM [11]. In our study, although we see a small but significant decrease in the ankle range compared to the control participants, we cannot assure if it is due to the limited plantar flexion or dorsiflexion. On the other hand, the deficit in hip flexion during the swing phase of gait cycle was also observed for other authors [21, 23] and would suggest a higher risk of falls in people with PD without training. Hip flexion occurs actively, facilitating the oscillation of the limb and helping to prevent foot drag during the swing phase. However, a poor hip flexion could favor a “shuffling gait” [24] which restrain people with PD from taking long and high steps. Despite these negative findings, we do not observe a decrease in the maximum hip extension reached on stance phase of gait cycle, which coincides with the observations of other studies during gait [5] both ON and OFF phase of the medication [23]. Finally, we observed a significant decrease in weight-acceptance and braking force and greater midstance force of the PDG compared to the HG. The influence that gait velocity has on the braking force [25] and on vertical forces [26], could explain these differences with the healthy group. In fact, high speeds (1.1–1.4 m/s) generate a higher vertical force in the loading response phase and lower in the midstance than low speeds (.83 m/s). In other words, the PD participants in our study tended to homogenize the vertical force curve compared to the control participants, which has been previously evidenced in conditions with and without medication effects [27]. Additionally, the decreased braking capacity observed in some people with PD reflects impaired postural control during gait [28] and it has been pointed out that this could be due to nondopaminergic lesions.

The results of this study, however, are obtained from participants with normal cognitive status, so future studies are needed to establish how functional tasks, secondary to gait, could interfere in people with PD in advanced stages and cognitive impairment. Another limitation of the study is that we used only day-to-day tasks in the evaluation, consequently, it is possible that the interference on the gait is influenced by the amount of daily practice of the selected tasks. Further, regarding the statistics, we should take into consideration that the Bonferroni adjustments conducted in the *post-hoc* comparisons could lead to an increase of the type II error [29]. Moreover, we have not studied whether different aspects of gait could have different neural control, which would explain the larger impact it has on some variables than on others. Otherwise, the participants were assessed in their bare feet to avoid the heterogeneity in the damping provided by the different footwear of the participants and therefore, prevent any confusion by that potential variability in the calculation of the ground reaction forces. However, it could affect the external validity, since in daily life, walking is conducted with shoes.

On the contrary, we conducted several DT conditions trying to cover the assessment of gait interference by secondary tasks, as it happens in habitual environments with multiples cognitive and motor demands. To assure face validity, the tests included were agreed by all the researchers and aligned with previous studies in which gait assessment was conducted [4, 12]. However, we did not analyze the construct validity of the gait assessment procedure. Therefore, we could not explore the homogeneity of the variables included in the conditions. Future works should analyze the construct validity of the outcomes included in the study to ensure that the gait pattern construct is being assessed.

## Conclusion

In untrained participants with PD, verbal and motor secondary tasks affect gait adversely and significantly, while auditory and visual tasks interfere to a lesser extent. Untrained people with PD have a poorer gait performance than their healthy counterparts both for the spatiotemporal, kinematic and kinetic variables analyzed, with the exception of the maximum hip extension during the stance phase of the gait cycle.

## Supplementary information

**Additional file 1.**

## Data Availability

The data collected in this study are available from the corresponding author on reasonable request.

## Abbreviations

PD: Parkinson’s disease
DT: Dual-tasking
PDG: Group of people with Parkinson’s disease
HG: Group of healthy people
ST: Single task
PPgait: Performance of parkinsonian gait
SD: Standard deviation
CI: Confidence intervals
