# Supplementary material for: Interference of functional dual-tasks on gait in untrained people with Parkinson’s disease and healthy controls: a cross-sectional study
Source: BMC Musculoskelet Disord. 2020 Jun 22;21:396. doi: 10.1186/s12891-020-03431-x (PMC7310477; doi:10.1186/s12891-020-03431-x)
Supplement: Supplementary file 1 — Additional file 1. [file 12891_2020_3431_MOESM1_ESM.docx]

**Post-hoc results from two-factor mixed Multivariate analysis of variance on biomechanical outcomes Comparisons between conditions**

| **Pair wise comparisons** | | | | | | | | |
| --- | --- | --- | --- | --- | --- | --- | --- | --- |
| Outcomes | Groups | (I)Condition | (J)Conditions | Mean difference (I-J) | Typical error | Sig.^a^ | 95 % Confidence Interval for the difference^a^ | |
|  |  |  |  |  |  |  | Lower limit | Upper limit |
| Velocity | Healthy control | Single-task | Visual DT | ,073 | ,039 | ,690 | -,041 | ,186 |
|  |  |  | Verbal DT | ,151^*^ | ,025 | ,000 | ,080 | ,222 |
|  |  |  | Auditory DT | ,145^*^ | ,019 | ,000 | ,090 | ,201 |
|  |  |  | Motor DT | ,123^*^ | ,020 | ,000 | ,066 | ,180 |
|  |  | Visual DT | Single-task | -,073 | ,039 | ,690 | -,186 | ,041 |
|  |  |  | Verbal DT | ,079 | ,041 | ,558 | -,038 | ,196 |
|  |  |  | Auditory DT | ,073 | ,040 | ,700 | -,042 | ,187 |
|  |  |  | Motor DT | ,051 | ,041 | 1,000 | -,067 | ,169 |
|  |  | Verbal DT | Single-task | -,151^*^ | ,025 | ,000 | -,222 | -,080 |
|  |  |  | Visual DT | -,079 | ,041 | ,558 | -,196 | ,038 |
|  |  |  | Auditory DT | -,006 | ,019 | 1,000 | -,061 | ,049 |
|  |  |  | Motor DT | -,028 | ,027 | 1,000 | -,105 | ,049 |
|  |  | Auditory DT | Single-task | -,145^*^ | ,019 | ,000 | -,201 | -,090 |
|  |  |  | Visual DT | -,073 | ,040 | ,700 | -,187 | ,042 |
|  |  |  | Verbal DT | ,006 | ,019 | 1,000 | -,049 | ,061 |
|  |  |  | Motor DT | -,022 | ,020 | 1,000 | -,080 | ,036 |
|  |  | Motor DT | Single-task | -,123^*^ | ,020 | ,000 | -,180 | -,066 |
|  |  |  | Visual DT | -,051 | ,041 | 1,000 | -,169 | ,067 |
|  |  |  | Verbal DT | ,028 | ,027 | 1,000 | -,049 | ,105 |
|  |  |  | Auditory DT | ,022 | ,020 | 1,000 | -,036 | ,080 |
|  | Parkinson's disease group | Single-task | Visual DT | ,073 | ,041 | ,765 | -,045 | ,191 |
|  |  |  | Verbal DT | ,146^*^ | ,025 | ,000 | ,073 | ,220 |
|  |  |  | Auditory DT | ,119^*^ | ,020 | ,000 | ,062 | ,177 |
|  |  |  | Motor DT | ,148^*^ | ,021 | ,000 | ,088 | ,207 |
|  |  | Visual DT | Single-task | -,073 | ,041 | ,765 | -,191 | ,045 |
|  |  |  | Verbal DT | ,073 | ,042 | ,870 | -,049 | ,195 |
|  |  |  | Auditory DT | ,046 | ,041 | 1,000 | -,073 | ,165 |
|  |  |  | Motor DT | ,074 | ,042 | ,835 | -,048 | ,197 |
|  |  | Verbal DT | Single-task | -,146^*^ | ,025 | ,000 | -,220 | -,073 |
|  |  |  | Visual DT | -,073 | ,042 | ,870 | -,195 | ,049 |
|  |  |  | Auditory DT | -,027 | ,020 | 1,000 | -,084 | ,030 |
|  |  |  | Motor DT | ,001 | ,028 | 1,000 | -,078 | ,081 |
|  |  | Auditory DT | Single-task | -,119^*^ | ,020 | ,000 | -,177 | -,062 |
|  |  |  | Visual DT | -,046 | ,041 | 1,000 | -,165 | ,073 |
|  |  |  | Verbal DT | ,027 | ,020 | 1,000 | -,030 | ,084 |
|  |  |  | Motor DT | ,028 | ,021 | 1,000 | -,032 | ,088 |
|  |  | Motor DT | Single-task | -,148^*^ | ,021 | ,000 | -,207 | -,088 |
|  |  |  | Visual DT | -,074 | ,042 | ,835 | -,197 | ,048 |
|  |  |  | Verbal DT | -,001 | ,028 | 1,000 | -,081 | ,078 |
|  |  |  | Auditory DT | -,028 | ,021 | 1,000 | -,088 | ,032 |
| Stride length | Healthy control | Single-task | Visual DT | ,070^*^ | ,021 | ,012 | ,010 | ,130 |
|  |  |  | Verbal DT | ,108^*^ | ,018 | ,000 | ,058 | ,159 |
|  |  |  | Auditory DT | ,091^*^ | ,020 | ,000 | ,034 | ,149 |
|  |  |  | Motor DT | ,097^*^ | ,029 | ,012 | ,013 | ,181 |
|  |  | Visual DT | Single-task | -,070^*^ | ,021 | ,012 | -,130 | -,010 |
|  |  |  | Verbal DT | ,038 | ,018 | ,402 | -,015 | ,091 |
|  |  |  | Auditory DT | ,021 | ,020 | 1,000 | -,038 | ,080 |
|  |  |  | Motor DT | ,027 | ,028 | 1,000 | -,053 | ,107 |
|  |  | Verbal DT | Single-task | -,108^*^ | ,018 | ,000 | -,159 | -,058 |
|  |  |  | Visual DT | -,038 | ,018 | ,402 | -,091 | ,015 |
|  |  |  | Auditory DT | -,017 | ,016 | 1,000 | -,064 | ,029 |
|  |  |  | Motor DT | -,011 | ,032 | 1,000 | -,103 | ,081 |
|  |  | Auditory DT | Single-task | -,091^*^ | ,020 | ,000 | -,149 | -,034 |
|  |  |  | Visual DT | -,021 | ,020 | 1,000 | -,080 | ,038 |
|  |  |  | Verbal DT | ,017 | ,016 | 1,000 | -,029 | ,064 |
|  |  |  | Motor DT | ,006 | ,029 | 1,000 | -,079 | ,091 |
|  |  | Motor DT | Single-task | -,097^*^ | ,029 | ,012 | -,181 | -,013 |
|  |  |  | Visual DT | -,027 | ,028 | 1,000 | -,107 | ,053 |
|  |  |  | Verbal DT | ,011 | ,032 | 1,000 | -,081 | ,103 |
|  |  |  | Auditory DT | -,006 | ,029 | 1,000 | -,091 | ,079 |
|  | Parkinson's disease group | Single-task | Visual DT | ,078^*^ | ,022 | ,006 | ,015 | ,140 |
|  |  |  | Verbal DT | ,132^*^ | ,018 | ,000 | ,079 | ,184 |
|  |  |  | Auditory DT | ,094^*^ | ,021 | ,000 | ,034 | ,153 |
|  |  |  | Motor DT | ,167^*^ | ,030 | ,000 | ,080 | ,253 |
|  |  | Visual DT | Single-task | -,078^*^ | ,022 | ,006 | -,140 | -,015 |
|  |  |  | Verbal DT | ,054 | ,019 | ,057 | -,001 | ,109 |
|  |  |  | Auditory DT | ,016 | ,021 | 1,000 | -,045 | ,077 |
|  |  |  | Motor DT | ,089^*^ | ,029 | ,028 | ,006 | ,172 |
|  |  | Verbal DT | Single-task | -,132^*^ | ,018 | ,000 | -,184 | -,079 |
|  |  |  | Visual DT | -,054 | ,019 | ,057 | -,109 | ,001 |
|  |  |  | Auditory DT | -,038 | ,017 | ,255 | -,086 | ,010 |
|  |  |  | Motor DT | ,035 | ,033 | 1,000 | -,060 | ,130 |
|  |  | Auditory DT | Single-task | -,094^*^ | ,021 | ,000 | -,153 | -,034 |
|  |  |  | Visual DT | -,016 | ,021 | 1,000 | -,077 | ,045 |
|  |  |  | Verbal DT | ,038 | ,017 | ,255 | -,010 | ,086 |
|  |  |  | Motor DT | ,073 | ,031 | ,195 | -,015 | ,161 |
|  |  | Motor DT | Single-task | -,167^*^ | ,030 | ,000 | -,253 | -,080 |
|  |  |  | Visual DT | -,089^*^ | ,029 | ,028 | -,172 | -,006 |
|  |  |  | Verbal DT | -,035 | ,033 | 1,000 | -,130 | ,060 |
|  |  |  | Auditory DT | -,073 | ,031 | ,195 | -,161 | ,015 |
| Cadence | Healthy control | Single-task | Visual DT | 4,052 | 1,829 | ,296 | -1,228 | 9,331 |
|  |  |  | Verbal DT | 7,449^*^ | 1,981 | ,003 | 1,730 | 13,167 |
|  |  |  | Auditory DT | 6,883^*^ | 2,125 | ,017 | ,751 | 13,015 |
|  |  |  | Motor DT | 4,430 | 1,863 | ,198 | -,946 | 9,806 |
|  |  | Visual DT | Single-task | -4,052 | 1,829 | ,296 | -9,331 | 1,228 |
|  |  |  | Verbal DT | 3,397 | 2,355 | 1,000 | -3,398 | 10,192 |
|  |  |  | Auditory DT | 2,832 | 2,548 | 1,000 | -4,522 | 10,185 |
|  |  |  | Motor DT | ,378 | 2,076 | 1,000 | -5,613 | 6,369 |
|  |  | Verbal DT | Single-task | -7,449^*^ | 1,981 | ,003 | -13,167 | -1,730 |
|  |  |  | Visual DT | -3,397 | 2,355 | 1,000 | -10,192 | 3,398 |
|  |  |  | Auditory DT | -,565 | 1,177 | 1,000 | -3,964 | 2,833 |
|  |  |  | Motor DT | -3,019 | 2,497 | 1,000 | -10,226 | 4,189 |
|  |  | Auditory DT | Single-task | -6,883^*^ | 2,125 | ,017 | -13,015 | -,751 |
|  |  |  | Visual DT | -2,832 | 2,548 | 1,000 | -10,185 | 4,522 |
|  |  |  | Verbal DT | ,565 | 1,177 | 1,000 | -2,833 | 3,964 |
|  |  |  | Motor DT | -2,453 | 2,708 | 1,000 | -10,268 | 5,361 |
|  |  | Motor DT | Single-task | -4,430 | 1,863 | ,198 | -9,806 | ,946 |
|  |  |  | Visual DT | -,378 | 2,076 | 1,000 | -6,369 | 5,613 |
|  |  |  | Verbal DT | 3,019 | 2,497 | 1,000 | -4,189 | 10,226 |
|  |  |  | Auditory DT | 2,453 | 2,708 | 1,000 | -5,361 | 10,268 |
|  | Parkinson's disease group | Single-task | Visual DT | ,353 | 1,897 | 1,000 | -5,120 | 5,827 |
|  |  |  | Verbal DT | 4,806 | 2,054 | ,218 | -1,123 | 10,735 |
|  |  |  | Auditory DT | 2,295 | 2,203 | 1,000 | -4,063 | 8,653 |
|  |  |  | Motor DT | 2,529 | 1,931 | 1,000 | -3,045 | 8,103 |
|  |  | Visual DT | Single-task | -,353 | 1,897 | 1,000 | -5,827 | 5,120 |
|  |  |  | Verbal DT | 4,453 | 2,441 | ,719 | -2,593 | 11,498 |
|  |  |  | Auditory DT | 1,941 | 2,642 | 1,000 | -5,683 | 9,566 |
|  |  |  | Motor DT | 2,176 | 2,152 | 1,000 | -4,036 | 8,388 |
|  |  | Verbal DT | Single-task | -4,806 | 2,054 | ,218 | -10,735 | 1,123 |
|  |  |  | Visual DT | -4,453 | 2,441 | ,719 | -11,498 | 2,593 |
|  |  |  | Auditory DT | -2,511 | 1,221 | ,429 | -6,035 | 1,012 |
|  |  |  | Motor DT | -2,277 | 2,589 | 1,000 | -9,750 | 5,196 |
|  |  | Auditory DT | Single-task | -2,295 | 2,203 | 1,000 | -8,653 | 4,063 |
|  |  |  | Visual DT | -1,941 | 2,642 | 1,000 | -9,566 | 5,683 |
|  |  |  | Verbal DT | 2,511 | 1,221 | ,429 | -1,012 | 6,035 |
|  |  |  | Motor DT | ,234 | 2,808 | 1,000 | -7,868 | 8,337 |
|  |  | Motor DT | Single-task | -2,529 | 1,931 | 1,000 | -8,103 | 3,045 |
|  |  |  | Visual DT | -2,176 | 2,152 | 1,000 | -8,388 | 4,036 |
|  |  |  | Verbal DT | 2,277 | 2,589 | 1,000 | -5,196 | 9,750 |
|  |  |  | Auditory DT | -,234 | 2,808 | 1,000 | -8,337 | 7,868 |
| Double support time | Healthy control | Single-task | Visual DT | -1,566^*^ | ,417 | ,003 | -2,770 | -,362 |
|  |  |  | Verbal DT | -1,919^*^ | ,490 | ,002 | -3,335 | -,504 |
|  |  |  | Auditory DT | -2,250^*^ | ,328 | ,000 | -3,196 | -1,304 |
|  |  |  | Motor DT | -2,416^*^ | ,461 | ,000 | -3,746 | -1,085 |
|  |  | Visual DT | Single-task | 1,566^*^ | ,417 | ,003 | ,362 | 2,770 |
|  |  |  | Verbal DT | -,353 | ,476 | 1,000 | -1,726 | 1,020 |
|  |  |  | Auditory DT | -,684 | ,442 | 1,000 | -1,959 | ,591 |
|  |  |  | Motor DT | -,850 | ,528 | 1,000 | -2,373 | ,674 |
|  |  | Verbal DT | Single-task | 1,919^*^ | ,490 | ,002 | ,504 | 3,335 |
|  |  |  | Visual DT | ,353 | ,476 | 1,000 | -1,020 | 1,726 |
|  |  |  | Auditory DT | -,331 | ,393 | 1,000 | -1,466 | ,804 |
|  |  |  | Motor DT | -,496 | ,532 | 1,000 | -2,030 | 1,038 |
|  |  | Auditory DT | Single-task | 2,250^*^ | ,328 | ,000 | 1,304 | 3,196 |
|  |  |  | Visual DT | ,684 | ,442 | 1,000 | -,591 | 1,959 |
|  |  |  | Verbal DT | ,331 | ,393 | 1,000 | -,804 | 1,466 |
|  |  |  | Motor DT | -,165 | ,502 | 1,000 | -1,616 | 1,285 |
|  |  | Motor DT | Single-task | 2,416^*^ | ,461 | ,000 | 1,085 | 3,746 |
|  |  |  | Visual DT | ,850 | ,528 | 1,000 | -,674 | 2,373 |
|  |  |  | Verbal DT | ,496 | ,532 | 1,000 | -1,038 | 2,030 |
|  |  |  | Auditory DT | ,165 | ,502 | 1,000 | -1,285 | 1,616 |
|  | Parkinson's disease group | Single-task | Visual DT | -2,148^*^ | ,433 | ,000 | -3,397 | -,899 |
|  |  |  | Verbal DT | -3,788^*^ | ,508 | ,000 | -5,255 | -2,321 |
|  |  |  | Auditory DT | -2,309^*^ | ,340 | ,000 | -3,289 | -1,328 |
|  |  |  | Motor DT | -3,197^*^ | ,478 | ,000 | -4,577 | -1,818 |
|  |  | Visual DT | Single-task | 2,148^*^ | ,433 | ,000 | ,899 | 3,397 |
|  |  |  | Verbal DT | -1,640^*^ | ,493 | ,013 | -3,064 | -,216 |
|  |  |  | Auditory DT | -,160 | ,458 | 1,000 | -1,483 | 1,162 |
|  |  |  | Motor DT | -1,049 | ,547 | ,588 | -2,629 | ,530 |
|  |  | Verbal DT | Single-task | 3,788^*^ | ,508 | ,000 | 2,321 | 5,255 |
|  |  |  | Visual DT | 1,640^*^ | ,493 | ,013 | ,216 | 3,064 |
|  |  |  | Auditory DT | 1,479^*^ | ,408 | ,005 | ,303 | 2,656 |
|  |  |  | Motor DT | ,591 | ,551 | 1,000 | -1,000 | 2,181 |
|  |  | Auditory DT | Single-task | 2,309^*^ | ,340 | ,000 | 1,328 | 3,289 |
|  |  |  | Visual DT | ,160 | ,458 | 1,000 | -1,162 | 1,483 |
|  |  |  | Verbal DT | -1,479^*^ | ,408 | ,005 | -2,656 | -,303 |
|  |  |  | Motor DT | -,889 | ,521 | ,919 | -2,392 | ,615 |
|  |  | Motor DT | Single-task | 3,197^*^ | ,478 | ,000 | 1,818 | 4,577 |
|  |  |  | Visual DT | 1,049 | ,547 | ,588 | -,530 | 2,629 |
|  |  |  | Verbal DT | -,591 | ,551 | 1,000 | -2,181 | 1,000 |
|  |  |  | Auditory DT | ,889 | ,521 | ,919 | -,615 | 2,392 |
| Ankle range | Healthy control | Single-task | Visual DT | ,832 | ,390 | ,360 | -,294 | 1,957 |
|  |  |  | Verbal DT | ,494 | ,350 | 1,000 | -,518 | 1,505 |
|  |  |  | Auditory DT | ,186 | ,529 | 1,000 | -1,341 | 1,712 |
|  |  |  | Motor DT | ,198 | ,479 | 1,000 | -1,183 | 1,579 |
|  |  | Visual DT | Single-task | -,832 | ,390 | ,360 | -1,957 | ,294 |
|  |  |  | Verbal DT | -,338 | ,388 | 1,000 | -1,457 | ,781 |
|  |  |  | Auditory DT | -,646 | ,578 | 1,000 | -2,314 | 1,022 |
|  |  |  | Motor DT | -,633 | ,440 | 1,000 | -1,903 | ,636 |
|  |  | Verbal DT | Single-task | -,494 | ,350 | 1,000 | -1,505 | ,518 |
|  |  |  | Visual DT | ,338 | ,388 | 1,000 | -,781 | 1,457 |
|  |  |  | Auditory DT | -,308 | ,506 | 1,000 | -1,768 | 1,152 |
|  |  |  | Motor DT | -,296 | ,426 | 1,000 | -1,525 | ,933 |
|  |  | Auditory DT | Single-task | -,186 | ,529 | 1,000 | -1,712 | 1,341 |
|  |  |  | Visual DT | ,646 | ,578 | 1,000 | -1,022 | 2,314 |
|  |  |  | Verbal DT | ,308 | ,506 | 1,000 | -1,152 | 1,768 |
|  |  |  | Motor DT | ,012 | ,574 | 1,000 | -1,643 | 1,668 |
|  |  | Motor DT | Single-task | -,198 | ,479 | 1,000 | -1,579 | 1,183 |
|  |  |  | Visual DT | ,633 | ,440 | 1,000 | -,636 | 1,903 |
|  |  |  | Verbal DT | ,296 | ,426 | 1,000 | -,933 | 1,525 |
|  |  |  | Auditory DT | -,012 | ,574 | 1,000 | -1,668 | 1,643 |
|  | Parkinson's disease group | Single-task | Visual DT | 1,170^*^ | ,404 | ,049 | ,003 | 2,336 |
|  |  |  | Verbal DT | 1,567^*^ | ,363 | ,000 | ,518 | 2,615 |
|  |  |  | Auditory DT | -,081 | ,548 | 1,000 | -1,664 | 1,501 |
|  |  |  | Motor DT | 2,029^*^ | ,496 | ,001 | ,596 | 3,461 |
|  |  | Visual DT | Single-task | -1,170^*^ | ,404 | ,049 | -2,336 | -,003 |
|  |  |  | Verbal DT | ,397 | ,402 | 1,000 | -,763 | 1,557 |
|  |  |  | Auditory DT | -1,251 | ,599 | ,399 | -2,981 | ,478 |
|  |  |  | Motor DT | ,859 | ,456 | ,633 | -,458 | 2,175 |
|  |  | Verbal DT | Single-task | -1,567^*^ | ,363 | ,000 | -2,615 | -,518 |
|  |  |  | Visual DT | -,397 | ,402 | 1,000 | -1,557 | ,763 |
|  |  |  | Auditory DT | -1,648^*^ | ,525 | ,023 | -3,162 | -,134 |
|  |  |  | Motor DT | ,462 | ,442 | 1,000 | -,812 | 1,736 |
|  |  | Auditory DT | Single-task | ,081 | ,548 | 1,000 | -1,501 | 1,664 |
|  |  |  | Visual DT | 1,251 | ,599 | ,399 | -,478 | 2,981 |
|  |  |  | Verbal DT | 1,648^*^ | ,525 | ,023 | ,134 | 3,162 |
|  |  |  | Motor DT | 2,110^*^ | ,595 | ,006 | ,394 | 3,826 |
|  |  | Motor DT | Single-task | -2,029^*^ | ,496 | ,001 | -3,461 | -,596 |
|  |  |  | Visual DT | -,859 | ,456 | ,633 | -2,175 | ,458 |
|  |  |  | Verbal DT | -,462 | ,442 | 1,000 | -1,736 | ,812 |
|  |  |  | Auditory DT | -2,110^*^ | ,595 | ,006 | -3,826 | -,394 |
| Hip extension | Healthy control | Single-task | Visual DT | ,281 | ,511 | 1,000 | -1,193 | 1,755 |
|  |  |  | Verbal DT | ,057 | ,502 | 1,000 | -1,391 | 1,505 |
|  |  |  | Auditory DT | -,137 | ,482 | 1,000 | -1,528 | 1,254 |
|  |  |  | Motor DT | -,222 | ,548 | 1,000 | -1,803 | 1,360 |
|  |  | Visual DT | Single-task | -,281 | ,511 | 1,000 | -1,755 | 1,193 |
|  |  |  | Verbal DT | -,224 | ,318 | 1,000 | -1,140 | ,693 |
|  |  |  | Auditory DT | -,418 | ,305 | 1,000 | -1,297 | ,462 |
|  |  |  | Motor DT | -,503 | ,413 | 1,000 | -1,695 | ,690 |
|  |  | Verbal DT | Single-task | -,057 | ,502 | 1,000 | -1,505 | 1,391 |
|  |  |  | Visual DT | ,224 | ,318 | 1,000 | -,693 | 1,140 |
|  |  |  | Auditory DT | -,194 | ,259 | 1,000 | -,941 | ,553 |
|  |  |  | Motor DT | -,279 | ,283 | 1,000 | -1,096 | ,538 |
|  |  | Auditory DT | Single-task | ,137 | ,482 | 1,000 | -1,254 | 1,528 |
|  |  |  | Visual DT | ,418 | ,305 | 1,000 | -,462 | 1,297 |
|  |  |  | Verbal DT | ,194 | ,259 | 1,000 | -,553 | ,941 |
|  |  |  | Motor DT | -,085 | ,315 | 1,000 | -,994 | ,825 |
|  |  | Motor DT | Single-task | ,222 | ,548 | 1,000 | -1,360 | 1,803 |
|  |  |  | Visual DT | ,503 | ,413 | 1,000 | -,690 | 1,695 |
|  |  |  | Verbal DT | ,279 | ,283 | 1,000 | -,538 | 1,096 |
|  |  |  | Auditory DT | ,085 | ,315 | 1,000 | -,825 | ,994 |
|  | Parkinson's disease group | Single-task | Visual DT | -,548 | ,530 | 1,000 | -2,076 | ,980 |
|  |  |  | Verbal DT | -,852 | ,520 | 1,000 | -2,354 | ,649 |
|  |  |  | Auditory DT | -,962 | ,500 | ,575 | -2,404 | ,479 |
|  |  |  | Motor DT | -1,330 | ,568 | ,216 | -2,970 | ,309 |
|  |  | Visual DT | Single-task | ,548 | ,530 | 1,000 | -,980 | 2,076 |
|  |  |  | Verbal DT | -,305 | ,329 | 1,000 | -1,255 | ,646 |
|  |  |  | Auditory DT | -,415 | ,316 | 1,000 | -1,327 | ,497 |
|  |  |  | Motor DT | -,783 | ,429 | ,714 | -2,019 | ,454 |
|  |  | Verbal DT | Single-task | ,852 | ,520 | 1,000 | -,649 | 2,354 |
|  |  |  | Visual DT | ,305 | ,329 | 1,000 | -,646 | 1,255 |
|  |  |  | Auditory DT | -,110 | ,268 | 1,000 | -,884 | ,664 |
|  |  |  | Motor DT | -,478 | ,293 | 1,000 | -1,325 | ,369 |
|  |  | Auditory DT | Single-task | ,962 | ,500 | ,575 | -,479 | 2,404 |
|  |  |  | Visual DT | ,415 | ,316 | 1,000 | -,497 | 1,327 |
|  |  |  | Verbal DT | ,110 | ,268 | 1,000 | -,664 | ,884 |
|  |  |  | Motor DT | -,368 | ,327 | 1,000 | -1,311 | ,575 |
|  |  | Motor DT | Single-task | 1,330 | ,568 | ,216 | -,309 | 2,970 |
|  |  |  | Visual DT | ,783 | ,429 | ,714 | -,454 | 2,019 |
|  |  |  | Verbal DT | ,478 | ,293 | 1,000 | -,369 | 1,325 |
|  |  |  | Auditory DT | ,368 | ,327 | 1,000 | -,575 | 1,311 |
| Hip flexion | Healthy control | Single-task | Visual DT | 1,771^*^ | ,561 | ,022 | ,152 | 3,390 |
|  |  |  | Verbal DT | 1,840^*^ | ,421 | ,000 | ,626 | 3,055 |
|  |  |  | Auditory DT | 2,118^*^ | ,471 | ,000 | ,757 | 3,478 |
|  |  |  | Motor DT | 2,184^*^ | ,418 | ,000 | ,978 | 3,390 |
|  |  | Visual DT | Single-task | -1,771^*^ | ,561 | ,022 | -3,390 | -,152 |
|  |  |  | Verbal DT | ,070 | ,524 | 1,000 | -1,442 | 1,581 |
|  |  |  | Auditory DT | ,347 | ,495 | 1,000 | -1,083 | 1,776 |
|  |  |  | Motor DT | ,413 | ,493 | 1,000 | -1,010 | 1,836 |
|  |  | Verbal DT | Single-task | -1,840^*^ | ,421 | ,000 | -3,055 | -,626 |
|  |  |  | Visual DT | -,070 | ,524 | 1,000 | -1,581 | 1,442 |
|  |  |  | Auditory DT | ,277 | ,303 | 1,000 | -,598 | 1,152 |
|  |  |  | Motor DT | ,343 | ,458 | 1,000 | -,977 | 1,664 |
|  |  | Auditory DT | Single-task | -2,118^*^ | ,471 | ,000 | -3,478 | -,757 |
|  |  |  | Visual DT | -,347 | ,495 | 1,000 | -1,776 | 1,083 |
|  |  |  | Verbal DT | -,277 | ,303 | 1,000 | -1,152 | ,598 |
|  |  |  | Motor DT | ,066 | ,442 | 1,000 | -1,211 | 1,343 |
|  |  | Motor DT | Single-task | -2,184^*^ | ,418 | ,000 | -3,390 | -,978 |
|  |  |  | Visual DT | -,413 | ,493 | 1,000 | -1,836 | 1,010 |
|  |  |  | Verbal DT | -,343 | ,458 | 1,000 | -1,664 | ,977 |
|  |  |  | Auditory DT | -,066 | ,442 | 1,000 | -1,343 | 1,211 |
|  | Parkinson's disease group | Single-task | Visual DT | ,541 | ,582 | 1,000 | -1,137 | 2,220 |
|  |  |  | Verbal DT | 1,437^*^ | ,436 | ,015 | ,178 | 2,696 |
|  |  |  | Auditory DT | ,623 | ,489 | 1,000 | -,787 | 2,034 |
|  |  |  | Motor DT | 1,220 | ,433 | ,061 | -,030 | 2,471 |
|  |  | Visual DT | Single-task | -,541 | ,582 | 1,000 | -2,220 | 1,137 |
|  |  |  | Verbal DT | ,895 | ,543 | 1,000 | -,671 | 2,462 |
|  |  |  | Auditory DT | ,082 | ,514 | 1,000 | -1,400 | 1,564 |
|  |  |  | Motor DT | ,679 | ,511 | 1,000 | -,797 | 2,154 |
|  |  | Verbal DT | Single-task | -1,437^*^ | ,436 | ,015 | -2,696 | -,178 |
|  |  |  | Visual DT | -,895 | ,543 | 1,000 | -2,462 | ,671 |
|  |  |  | Auditory DT | -,814 | ,314 | ,114 | -1,721 | ,093 |
|  |  |  | Motor DT | -,217 | ,475 | 1,000 | -1,586 | 1,153 |
|  |  | Auditory DT | Single-task | -,623 | ,489 | 1,000 | -2,034 | ,787 |
|  |  |  | Visual DT | -,082 | ,514 | 1,000 | -1,564 | 1,400 |
|  |  |  | Verbal DT | ,814 | ,314 | ,114 | -,093 | 1,721 |
|  |  |  | Motor DT | ,597 | ,459 | 1,000 | -,727 | 1,921 |
|  |  | Motor DT | Single-task | -1,220 | ,433 | ,061 | -2,471 | ,030 |
|  |  |  | Visual DT | -,679 | ,511 | 1,000 | -2,154 | ,797 |
|  |  |  | Verbal DT | ,217 | ,475 | 1,000 | -1,153 | 1,586 |
|  |  |  | Auditory DT | -,597 | ,459 | 1,000 | -1,921 | ,727 |
| Weight-acceptance force | Healthy control | Single-task | Visual DT | ,278 | ,098 | ,058 | -,005 | ,562 |
|  |  |  | Verbal DT | ,423^*^ | ,079 | ,000 | ,194 | ,653 |
|  |  |  | Auditory DT | ,446^*^ | ,086 | ,000 | ,198 | ,694 |
|  |  |  | Motor DT | ,289^*^ | ,096 | ,034 | ,012 | ,566 |
|  |  | Visual DT | Single-task | -,278 | ,098 | ,058 | -,562 | ,005 |
|  |  |  | Verbal DT | ,145 | ,085 | ,928 | -,101 | ,390 |
|  |  |  | Auditory DT | ,167 | ,086 | ,560 | -,082 | ,417 |
|  |  |  | Motor DT | ,011 | ,108 | 1,000 | -,300 | ,322 |
|  |  | Verbal DT | Single-task | -,423^*^ | ,079 | ,000 | -,653 | -,194 |
|  |  |  | Visual DT | -,145 | ,085 | ,928 | -,390 | ,101 |
|  |  |  | Auditory DT | ,023 | ,045 | 1,000 | -,109 | ,154 |
|  |  |  | Motor DT | -,134 | ,064 | ,406 | -,319 | ,052 |
|  |  | Auditory DT | Single-task | -,446^*^ | ,086 | ,000 | -,694 | -,198 |
|  |  |  | Visual DT | -,167 | ,086 | ,560 | -,417 | ,082 |
|  |  |  | Verbal DT | -,023 | ,045 | 1,000 | -,154 | ,109 |
|  |  |  | Motor DT | -,157 | ,078 | ,477 | -,381 | ,068 |
|  |  | Motor DT | Single-task | -,289^*^ | ,096 | ,034 | -,566 | -,012 |
|  |  |  | Visual DT | -,011 | ,108 | 1,000 | -,322 | ,300 |
|  |  |  | Verbal DT | ,134 | ,064 | ,406 | -,052 | ,319 |
|  |  |  | Auditory DT | ,157 | ,078 | ,477 | -,068 | ,381 |
|  | Parkinson's disease group | Single-task | Visual DT | ,099 | ,102 | 1,000 | -,194 | ,393 |
|  |  |  | Verbal DT | ,121 | ,082 | 1,000 | -,116 | ,359 |
|  |  |  | Auditory DT | ,124 | ,089 | 1,000 | -,133 | ,381 |
|  |  |  | Motor DT | ,109 | ,100 | 1,000 | -,178 | ,396 |
|  |  | Visual DT | Single-task | -,099 | ,102 | 1,000 | -,393 | ,194 |
|  |  |  | Verbal DT | ,022 | ,088 | 1,000 | -,233 | ,277 |
|  |  |  | Auditory DT | ,025 | ,090 | 1,000 | -,233 | ,283 |
|  |  |  | Motor DT | ,009 | ,112 | 1,000 | -,313 | ,332 |
|  |  | Verbal DT | Single-task | -,121 | ,082 | 1,000 | -,359 | ,116 |
|  |  |  | Visual DT | -,022 | ,088 | 1,000 | -,277 | ,233 |
|  |  |  | Auditory DT | ,003 | ,047 | 1,000 | -,133 | ,139 |
|  |  |  | Motor DT | -,012 | ,067 | 1,000 | -,205 | ,180 |
|  |  | Auditory DT | Single-task | -,124 | ,089 | 1,000 | -,381 | ,133 |
|  |  |  | Visual DT | -,025 | ,090 | 1,000 | -,283 | ,233 |
|  |  |  | Verbal DT | -,003 | ,047 | 1,000 | -,139 | ,133 |
|  |  |  | Motor DT | -,016 | ,081 | 1,000 | -,248 | ,217 |
|  |  | Motor DT | Single-task | -,109 | ,100 | 1,000 | -,396 | ,178 |
|  |  |  | Visual DT | -,009 | ,112 | 1,000 | -,332 | ,313 |
|  |  |  | Verbal DT | ,012 | ,067 | 1,000 | -,180 | ,205 |
|  |  |  | Auditory DT | ,016 | ,081 | 1,000 | -,217 | ,248 |
| Midstance force | Healthy control | Single-task | Visual DT | -,189 | ,073 | ,114 | -,400 | ,022 |
|  |  |  | Verbal DT | -,475^*^ | ,079 | ,000 | -,702 | -,248 |
|  |  |  | Auditory DT | -,551^*^ | ,080 | ,000 | -,783 | -,319 |
|  |  |  | Motor DT | -,398^*^ | ,081 | ,000 | -,633 | -,164 |
|  |  | Visual DT | Single-task | ,189 | ,073 | ,114 | -,022 | ,400 |
|  |  |  | Verbal DT | -,286^*^ | ,063 | ,000 | -,469 | -,104 |
|  |  |  | Auditory DT | -,362^*^ | ,061 | ,000 | -,538 | -,187 |
|  |  |  | Motor DT | -,209^*^ | ,067 | ,025 | -,403 | -,016 |
|  |  | Verbal DT | Single-task | ,475^*^ | ,079 | ,000 | ,248 | ,702 |
|  |  |  | Visual DT | ,286^*^ | ,063 | ,000 | ,104 | ,469 |
|  |  |  | Auditory DT | -,076 | ,052 | 1,000 | -,226 | ,074 |
|  |  |  | Motor DT | ,077 | ,058 | 1,000 | -,090 | ,243 |
|  |  | Auditory DT | Single-task | ,551^*^ | ,080 | ,000 | ,319 | ,783 |
|  |  |  | Visual DT | ,362^*^ | ,061 | ,000 | ,187 | ,538 |
|  |  |  | Verbal DT | ,076 | ,052 | 1,000 | -,074 | ,226 |
|  |  |  | Motor DT | ,153 | ,064 | ,192 | -,032 | ,338 |
|  |  | Motor DT | Single-task | ,398^*^ | ,081 | ,000 | ,164 | ,633 |
|  |  |  | Visual DT | ,209^*^ | ,067 | ,025 | ,016 | ,403 |
|  |  |  | Verbal DT | -,077 | ,058 | 1,000 | -,243 | ,090 |
|  |  |  | Auditory DT | -,153 | ,064 | ,192 | -,338 | ,032 |
|  | Parkinson's disease group | Single-task | Visual DT | -,126 | ,076 | 1,000 | -,344 | ,093 |
|  |  |  | Verbal DT | -,353^*^ | ,082 | ,000 | -,588 | -,117 |
|  |  |  | Auditory DT | -,254^*^ | ,083 | ,032 | -,494 | -,013 |
|  |  |  | Motor DT | -,299^*^ | ,084 | ,007 | -,542 | -,056 |
|  |  | Visual DT | Single-task | ,126 | ,076 | 1,000 | -,093 | ,344 |
|  |  |  | Verbal DT | -,227^*^ | ,066 | ,009 | -,416 | -,038 |
|  |  |  | Auditory DT | -,128 | ,063 | ,455 | -,310 | ,054 |
|  |  |  | Motor DT | -,173 | ,069 | ,148 | -,374 | ,027 |
|  |  | Verbal DT | Single-task | ,353^*^ | ,082 | ,000 | ,117 | ,588 |
|  |  |  | Visual DT | ,227^*^ | ,066 | ,009 | ,038 | ,416 |
|  |  |  | Auditory DT | ,099 | ,054 | ,694 | -,056 | ,254 |
|  |  |  | Motor DT | ,054 | ,060 | 1,000 | -,119 | ,227 |
|  |  | Auditory DT | Single-task | ,254^*^ | ,083 | ,032 | ,013 | ,494 |
|  |  |  | Visual DT | ,128 | ,063 | ,455 | -,054 | ,310 |
|  |  |  | Verbal DT | -,099 | ,054 | ,694 | -,254 | ,056 |
|  |  |  | Motor DT | -,045 | ,066 | 1,000 | -,237 | ,146 |
|  |  | Motor DT | Single-task | ,299^*^ | ,084 | ,007 | ,056 | ,542 |
|  |  |  | Visual DT | ,173 | ,069 | ,148 | -,027 | ,374 |
|  |  |  | Verbal DT | -,054 | ,060 | 1,000 | -,227 | ,119 |
|  |  |  | Auditory DT | ,045 | ,066 | 1,000 | -,146 | ,237 |
| Braking force | Healthy control | Single-task | Visual DT | -,157^*^ | ,034 | ,000 | -,256 | -,057 |
|  |  |  | Verbal DT | -,185^*^ | ,042 | ,000 | -,306 | -,064 |
|  |  |  | Auditory DT | -,258^*^ | ,041 | ,000 | -,375 | -,140 |
|  |  |  | Motor DT | -,196^*^ | ,059 | ,012 | -,365 | -,027 |
|  |  | Visual DT | Single-task | ,157^*^ | ,034 | ,000 | ,057 | ,256 |
|  |  |  | Verbal DT | -,028 | ,042 | 1,000 | -,149 | ,092 |
|  |  |  | Auditory DT | -,101^*^ | ,034 | ,038 | -,199 | -,003 |
|  |  |  | Motor DT | -,039 | ,055 | 1,000 | -,198 | ,119 |
|  |  | Verbal DT | Single-task | ,185^*^ | ,042 | ,000 | ,064 | ,306 |
|  |  |  | Visual DT | ,028 | ,042 | 1,000 | -,092 | ,149 |
|  |  |  | Auditory DT | -,073 | ,035 | ,414 | -,174 | ,028 |
|  |  |  | Motor DT | -,011 | ,056 | 1,000 | -,173 | ,151 |
|  |  | Auditory DT | Single-task | ,258^*^ | ,041 | ,000 | ,140 | ,375 |
|  |  |  | Visual DT | ,101^*^ | ,034 | ,038 | ,003 | ,199 |
|  |  |  | Verbal DT | ,073 | ,035 | ,414 | -,028 | ,174 |
|  |  |  | Motor DT | ,062 | ,053 | 1,000 | -,090 | ,213 |
|  |  | Motor DT | Single-task | ,196^*^ | ,059 | ,012 | ,027 | ,365 |
|  |  |  | Visual DT | ,039 | ,055 | 1,000 | -,119 | ,198 |
|  |  |  | Verbal DT | ,011 | ,056 | 1,000 | -,151 | ,173 |
|  |  |  | Auditory DT | -,062 | ,053 | 1,000 | -,213 | ,090 |
|  | Parkinson's disease group | Single-task | Visual DT | -,145^*^ | ,036 | ,001 | -,248 | -,042 |
|  |  |  | Verbal DT | -,299^*^ | ,043 | ,000 | -,425 | -,174 |
|  |  |  | Auditory DT | -,176^*^ | ,042 | ,001 | -,297 | -,054 |
|  |  |  | Motor DT | -,223^*^ | ,061 | ,004 | -,398 | -,047 |
|  |  | Visual DT | Single-task | ,145^*^ | ,036 | ,001 | ,042 | ,248 |
|  |  |  | Verbal DT | -,155^*^ | ,043 | ,006 | -,280 | -,029 |
|  |  |  | Auditory DT | -,031 | ,035 | 1,000 | -,132 | ,070 |
|  |  |  | Motor DT | -,078 | ,057 | 1,000 | -,243 | ,087 |
|  |  | Verbal DT | Single-task | ,299^*^ | ,043 | ,000 | ,174 | ,425 |
|  |  |  | Visual DT | ,155^*^ | ,043 | ,006 | ,029 | ,280 |
|  |  |  | Auditory DT | ,124^*^ | ,036 | ,010 | ,019 | ,229 |
|  |  |  | Motor DT | ,077 | ,058 | 1,000 | -,091 | ,244 |
|  |  | Auditory DT | Single-task | ,176^*^ | ,042 | ,001 | ,054 | ,297 |
|  |  |  | Visual DT | ,031 | ,035 | 1,000 | -,070 | ,132 |
|  |  |  | Verbal DT | -,124^*^ | ,036 | ,010 | -,229 | -,019 |
|  |  |  | Motor DT | -,047 | ,055 | 1,000 | -,205 | ,110 |
|  |  | Motor DT | Single-task | ,223^*^ | ,061 | ,004 | ,047 | ,398 |
|  |  |  | Visual DT | ,078 | ,057 | 1,000 | -,087 | ,243 |
|  |  |  | Verbal DT | -,077 | ,058 | 1,000 | -,244 | ,091 |
|  |  |  | Auditory DT | ,047 | ,055 | 1,000 | -,110 | ,205 |
| Based on estimated marginal measures. | | | | | | | | |
| a. Fit for multiple comparisons: Bonferroni.  *. The mean difference is significant at ,05 level. | | | | | | | | |

**Post-hoc results from two-factor mixed Multivariate analysis of variance on biomechanical outcomes Comparisons between groups**

| **Pair wise comparisons** | | | | | | | | |
| --- | --- | --- | --- | --- | --- | --- | --- | --- |
| Outcomes | Conditions | (I)Groups | (J)Groups | Mean difference (I-J) | Typical error | Sig.^a^ | 95 % Confidence Interval for the difference ^a^ | |
|  |  |  |  |  |  |  | Lower limit | Upper limit |
| Velocity | Single-task | HG | PDG | ,215^*^ | ,037 | ,000 | ,142 | ,288 |
|  |  | PDG | HG | -,215^*^ | ,037 | ,000 | -,288 | -,142 |
|  | Visual DT | HG | PDG | ,216^*^ | ,044 | ,000 | ,128 | ,304 |
|  |  | PDG | HG | -,216^*^ | ,044 | ,000 | -,304 | -,128 |
|  | Verbal DT | HG | PDG | ,210^*^ | ,042 | ,000 | ,127 | ,293 |
|  |  | PDG | HG | -,210^*^ | ,042 | ,000 | -,293 | -,127 |
|  | Auditory DT | HG | PDG | ,189^*^ | ,039 | ,000 | ,111 | ,267 |
|  |  | PDG | HG | -,189^*^ | ,039 | ,000 | -,267 | -,111 |
|  | Motor DT | HG | PDG | ,239^*^ | ,042 | ,000 | ,155 | ,324 |
|  |  | PDG | HG | -,239^*^ | ,042 | ,000 | -,324 | -,155 |
| Stride length | Single-task | HG | PDG | ,171^*^ | ,038 | ,000 | ,095 | ,247 |
|  |  | PDG | HG | -,171^*^ | ,038 | ,000 | -,247 | -,095 |
|  | Visual DT | HG | PDG | ,178^*^ | ,041 | ,000 | ,097 | ,259 |
|  |  | PDG | HG | -,178^*^ | ,041 | ,000 | -,259 | -,097 |
|  | Verbal DT | HG | PDG | ,194^*^ | ,038 | ,000 | ,118 | ,270 |
|  |  | PDG | HG | -,194^*^ | ,038 | ,000 | -,270 | -,118 |
|  | Auditory DT | HG | PDG | ,173^*^ | ,039 | ,000 | ,095 | ,252 |
|  |  | PDG | HG | -,173^*^ | ,039 | ,000 | -,252 | -,095 |
|  | Motor DT | HG | PDG | ,240^*^ | ,053 | ,000 | ,136 | ,345 |
|  |  | PDG | HG | -,240^*^ | ,053 | ,000 | -,345 | -,136 |
| Cadence | Single-task | HG | PDG | 9,402^*^ | 1,990 | ,000 | 5,443 | 13,361 |
|  |  | PDG | HG | -9,402^*^ | 1,990 | ,000 | -13,361 | -5,443 |
|  | Visual DT | HG | PDG | 5,704 | 3,292 | ,087 | -,847 | 12,254 |
|  |  | PDG | HG | -5,704 | 3,292 | ,087 | -12,254 | ,847 |
|  | Verbal DT | HG | PDG | 6,759^*^ | 3,349 | ,047 | ,095 | 13,424 |
|  |  | PDG | HG | -6,759^*^ | 3,349 | ,047 | -13,424 | -,095 |
|  | Auditory DT | HG | PDG | 4,814 | 3,470 | ,169 | -2,091 | 11,718 |
|  |  | PDG | HG | -4,814 | 3,470 | ,169 | -11,718 | 2,091 |
|  | Motor DT | HG | PDG | 7,501^*^ | 3,354 | ,028 | ,827 | 14,176 |
|  |  | PDG | HG | -7,501^*^ | 3,354 | ,028 | -14,176 | -,827 |
| Double support time | Single-task | HG | PDG | -3,714^*^ | ,649 | ,000 | -5,005 | -2,424 |
|  |  | PDG | HG | 3,714^*^ | ,649 | ,000 | 2,424 | 5,005 |
|  | Visual DT | HG | PDG | -4,296^*^ | ,753 | ,000 | -5,794 | -2,798 |
|  |  | PDG | HG | 4,296^*^ | ,753 | ,000 | 2,798 | 5,794 |
|  | Verbal DT | HG | PDG | -5,583^*^ | ,921 | ,000 | -7,415 | -3,751 |
|  |  | PDG | HG | 5,583^*^ | ,921 | ,000 | 3,751 | 7,415 |
|  | Auditory DT | HG | PDG | -3,773^*^ | ,722 | ,000 | -5,210 | -2,335 |
|  |  | PDG | HG | 3,773^*^ | ,722 | ,000 | 2,335 | 5,210 |
|  | Motor DT | HG | PDG | -4,496^*^ | ,884 | ,000 | -6,255 | -2,737 |
|  |  | PDG | HG | 4,496^*^ | ,884 | ,000 | 2,737 | 6,255 |
| Ankle range | Single-task | HG | PDG | 1,917^*^ | ,846 | ,026 | ,234 | 3,599 |
|  |  | PDG | HG | -1,917^*^ | ,846 | ,026 | -3,599 | -,234 |
|  | Visual DT | HG | PDG | 2,255^*^ | ,861 | ,011 | ,541 | 3,969 |
|  |  | PDG | HG | -2,255^*^ | ,861 | ,011 | -3,969 | -,541 |
|  | Verbal DT | HG | PDG | 2,990^*^ | ,808 | ,000 | 1,383 | 4,596 |
|  |  | PDG | HG | -2,990^*^ | ,808 | ,000 | -4,596 | -1,383 |
|  | Auditory DT | HG | PDG | 1,649 | ,904 | ,072 | -,150 | 3,448 |
|  |  | PDG | HG | -1,649 | ,904 | ,072 | -3,448 | ,150 |
|  | Motor DT | HG | PDG | 3,747^*^ | ,757 | ,000 | 2,240 | 5,254 |
|  |  | PDG | HG | -3,747^*^ | ,757 | ,000 | -5,254 | -2,240 |
| Hip extension | Single-task | HG | PDG | ,273 | 1,079 | ,801 | -1,875 | 2,420 |
|  |  | PDG | HG | -,273 | 1,079 | ,801 | -2,420 | 1,875 |
|  | Visual DT | HG | PDG | -,556 | ,814 | ,496 | -2,175 | 1,063 |
|  |  | PDG | HG | ,556 | ,814 | ,496 | -1,063 | 2,175 |
|  | Verbal DT | HG | PDG | -,637 | ,845 | ,453 | -2,317 | 1,044 |
|  |  | PDG | HG | ,637 | ,845 | ,453 | -1,044 | 2,317 |
|  | Auditory DT | HG | PDG | -,553 | ,834 | ,510 | -2,213 | 1,107 |
|  |  | PDG | HG | ,553 | ,834 | ,510 | -1,107 | 2,213 |
|  | Motor DT | HG | PDG | -,836 | ,834 | ,319 | -2,495 | ,823 |
|  |  | PDG | HG | ,836 | ,834 | ,319 | -,823 | 2,495 |
| Hip flexion | Single-task | HG | PDG | 4,604^*^ | 1,606 | ,005 | 1,408 | 7,800 |
|  |  | PDG | HG | -4,604^*^ | 1,606 | ,005 | -7,800 | -1,408 |
|  | Visual DT | HG | PDG | 3,375^*^ | 1,535 | ,031 | ,321 | 6,428 |
|  |  | PDG | HG | -3,375^*^ | 1,535 | ,031 | -6,428 | -,321 |
|  | Verbal DT | HG | PDG | 4,201^*^ | 1,431 | ,004 | 1,354 | 7,047 |
|  |  | PDG | HG | -4,201^*^ | 1,431 | ,004 | -7,047 | -1,354 |
|  | Auditory DT | HG | PDG | 3,110^*^ | 1,442 | ,034 | ,241 | 5,978 |
|  |  | PDG | HG | -3,110^*^ | 1,442 | ,034 | -5,978 | -,241 |
|  | Motor DT | HG | PDG | 3,640^*^ | 1,492 | ,017 | ,671 | 6,610 |
|  |  | PDG | HG | -3,640^*^ | 1,492 | ,017 | -6,610 | -,671 |
| Weight-acceptance force | Single-task | HG | PDG | ,678^*^ | ,192 | ,001 | ,296 | 1,060 |
|  |  | PDG | HG | -,678^*^ | ,192 | ,001 | -1,060 | -,296 |
|  | Visual DT | HG | PDG | ,499^*^ | ,197 | ,013 | ,108 | ,891 |
|  |  | PDG | HG | -,499^*^ | ,197 | ,013 | -,891 | -,108 |
|  | Verbal DT | HG | PDG | ,376^*^ | ,138 | ,008 | ,103 | ,650 |
|  |  | PDG | HG | -,376^*^ | ,138 | ,008 | -,650 | -,103 |
|  | Auditory DT | HG | PDG | ,357^*^ | ,152 | ,021 | ,055 | ,659 |
|  |  | PDG | HG | -,357^*^ | ,152 | ,021 | -,659 | -,055 |
|  | Motor DT | HG | PDG | ,498^*^ | ,136 | ,000 | ,227 | ,769 |
|  |  | PDG | HG | -,498^*^ | ,136 | ,000 | -,769 | -,227 |
| Midstance force | Single-task | HG | PDG | -,777^*^ | ,159 | ,000 | -1,093 | -,462 |
|  |  | PDG | HG | ,777^*^ | ,159 | ,000 | ,462 | 1,093 |
|  | Visual DT | HG | PDG | -,714^*^ | ,161 | ,000 | -1,034 | -,394 |
|  |  | PDG | HG | ,714^*^ | ,161 | ,000 | ,394 | 1,034 |
|  | Verbal DT | HG | PDG | -,655^*^ | ,132 | ,000 | -,919 | -,391 |
|  |  | PDG | HG | ,655^*^ | ,132 | ,000 | ,391 | ,919 |
|  | Auditory DT | HG | PDG | -,480^*^ | ,136 | ,001 | -,751 | -,209 |
|  |  | PDG | HG | ,480^*^ | ,136 | ,001 | ,209 | ,751 |
|  | Motor DT | HG | PDG | -,678^*^ | ,141 | ,000 | -,958 | -,398 |
|  |  | PDG | HG | ,678^*^ | ,141 | ,000 | ,398 | ,958 |
| Braking force | Single-task | HG | PDG | -,355^*^ | ,088 | ,000 | -,529 | -,180 |
|  |  | PDG | HG | ,355^*^ | ,088 | ,000 | ,180 | ,529 |
|  | Visual DT | HG | PDG | -,343^*^ | ,085 | ,000 | -,512 | -,174 |
|  |  | PDG | HG | ,343^*^ | ,085 | ,000 | ,174 | ,512 |
|  | Verbal DT | HG | PDG | -,469^*^ | ,088 | ,000 | -,645 | -,293 |
|  |  | PDG | HG | ,469^*^ | ,088 | ,000 | ,293 | ,645 |
|  | Auditory DT | HG | PDG | -,273^*^ | ,078 | ,001 | -,428 | -,118 |
|  |  | PDG | HG | ,273^*^ | ,078 | ,001 | ,118 | ,428 |
|  | Motor DT | HG | PDG | -,381^*^ | ,058 | ,000 | -,497 | -,266 |
|  |  | PDG | HG | ,381^*^ | ,058 | ,000 | ,266 | ,497 |
| Based on estimated marginal measures. | | | | | | | | |
| a. Fit for multiple comparisons: Bonferroni.  *. The mean difference is significant at ,05 level.  HG: Healthy group. PDG: Parkinson's disease group. | | | | | | | | |
